# Effects of Department Type and Disability Status on Medical School Faculty Wellbeing

**DOI:** 10.1002/hsr2.70317

**Published:** 2025-01-09

**Authors:** Mohammed A. A. Abulela, Bethany Schowengerdt, Heather Dorr, Amanda Termuhlen, Kristina Krohn, Claudio Violato

**Affiliations:** ^1^ Department of Educational Psychology University of Minnesota Minneapolis Minnesota USA; ^2^ University of Minnesota Medical School Minneapolis Minnesota USA; ^3^ Research Methodology Consulting Center University of Minnesota Minneapolis Minnesota USA; ^4^ Department of Organizational Leadership, Policy, and Development University of Minnesota Minneapolis Minnesota USA

**Keywords:** association, burnout, disability, wellbeing, workload

## Abstract

**Purpose:**

Faculty wellbeing impacts student learning and is a priority among medical schools, especially as a counterbalance to growing burnout. Previous researchers found differences in burnout by sex and race among clinicians, but not for faculty with disabilities. Accordingly, the purpose was to test the association between faculty's wellbeing, burnout, and control over workload and investigate differences in wellbeing attributed to department type and ability status.

**Method:**

The authors developed and administered a comprehensive wellbeing survey to University of Minnesota Medical School faculty, of whom 703 provided complete responses. The authors conducted two‐way ANOVA followed by a post hoc analysis to test for differences in faculty wellbeing domains due to department type (basic sciences, nonsurgical, surgical, and two large departments of Medicine and Pediatrics) and disability status (yes, no). The authors also fitted a two‐way ordinal model since burnout frequency and control over workload were assessed by one ordinal item each.

**Results:**

Wellbeing domains were positively correlated with control over workload but negatively associated with burnout. Faculty with disabilities reported less support from their work environment and meeting of their basic needs. Department type had a statistically significant impact on faculty's sense of basic needs, respect, and contribution. Multiple comparisons revealed faculty in basic sciences departments had higher scores within basic needs compared to the departments of Medicine, Pediatrics, and surgical departments, who reported lower levels of respect as well. Results revealed department type and disability status affected the frequency of burnout, as faculty in basic sciences departments reported lower levels of burnout compared to other departments.

**Conclusions:**

Results support disaggregating wellbeing by department and ability status for targeted interventions due to differences‐ notably among faculty with disabilities and surgical departments‐ in their assessment of basic needs, work environment, respect, and contribution. Results suggest revisiting interventions in these domains to account for lower reported wellbeing.

## 1 Introduction

Burnout is a well‐known issue affecting physicians and the care they provide patients [1, 2], to the point where it is arguably a quality indicator for health care [3]. Burnout was initially defined for health professionals as a combination of symptoms that harm both workers' health and affect job performance. These symptoms include emotional exhaustion from continually confronting stressors, feeling detached from co‐workers and one's professional identity, as well as perceived inefficacy in one's role [4]. Clinical faculty, practicing physicians who teach in medical schools, experience burnout at the same level as nonfaculty physicians in the community [5, 6]. Health systems [7] and medical schools [8, 9] are attempting to shift the quality of healthcare by addressing wellbeing, intensively in the wake of the COVID‐19 pandemic. Ensuring the wellbeing of medical school faculty, including basic sciences faculty, is not only intrinsically beneficial, just, and potentially preventative of burnout but can also benefit medical students' learning [10] and professional development [11]. Yet, despite increased research on burnout in academic medicine [12] since the 1970s, interventions targeting wellbeing of medical school faculty have not consistently delivered on these potential benefits [13, 14]. Not enough is known about the interaction of factors connecting wellbeing to burnout in medical schools in the current climate to inform wellbeing initiatives [14]. Part of this issue is a lack of disaggregation of the concept of wellbeing itself [13, 15] and then targeting wellbeing measures and prioritizing interventions by faculty's backgrounds [6, 16]. To shift this paradigm, challenging the limitations of prevalent wellbeing models and their limited use in faculty affairs research is necessary to improve interventions for specific populations of academic medicine faculty. Specifically, we argue that mapping the wellbeing of faculty holistically, in the context of interrelations with colleagues and embeddedness in organizational dynamics, will provide more actionable guidance to prevent burnout. The next section provides an overview of the development of models to measure medical school faculty wellbeing.

### 1.1 Understanding Faculty's Wellbeing in an Organizational Context

Different models have been employed to study faculty wellbeing in academic medicine, moving towards a more collective conceptualization of individual's wellbeing within an organization and a profession. For instance, research on satisfaction in organizational studies led to a model of balancing job demands and faculty resources [17] adopted by the National Academy of Medicine [18]. This model focuses heavily on material resources and not on faculty agency. By contrast, models based on self‐determination theory [19] posit that wellbeing relies on how resources enable/disable faculty from acting on their goals via their fulfillment of three psychological needs. Outside of academic medicine, research on university faculty suggests perceived competence, perceived relatedness (intellectually and collegially to the organization), and autonomy to influence choices, each the effect of certain supports (e.g., from administration, for their research) to satisfaction [20].

Scholars have begun to develop models specific to medical education from phenomenological [14] and comparative qualitative research, which emphasizes the relatedness of faculty to other faculty, students, their administration, and policy, and the affective nature of perceptions of their work beyond perceived competence and autonomy [5]. For example, models such as vitality [21], resilience [13], and domains of wellbeing [15] emphasize the multifaceted, even hierarchical, nature of wellbeing and a person's embeddedness in social expectations, adversity, and moral responsibility. Shapiro et al. [15] drew from Maslow's hierarchy of needs to explain the interrelationships between (a) basics (i.e., access to food and time to eat, personal time), (b) safety, (i.e., job security, individual and workplace strategies for stress management, psychological safety), (c) respect (i.e., esteem by peers, supervisor, organization, the community at large) (d) appreciation (i.e., connection to the department, valued by organization and colleagues), and (e) contribution (i.e., adequate resources and opportunity to make the most of one's role, to provide inputs regarding institutional decisions). Shapiro et al. [15] argued that understanding any one domain, such as contribution, in isolation, or personally and not systemically limits institutional capacity to address burnout. This is similar to Vercio et al. [13], who, in addition, contended that using resilience, rather than wellbeing, accounts for the adversity that faculty and organizations face without assuming increased wellbeing decreases burnout. Both framings critique more individually focused models such as self‐determination. Yet, unlike vitality and resilience, Shapiro et al.'s domains account for the fundamentality of basic material needs.

### 1.2 Limitations of Organizational Research on Faculty Wellbeing in Medical Schools

Organizationally attuned models have not yet informed empirical research or instruments examining the connection between wellbeing and burnout in medical school faculty, despite the priority of medical schools to “balance the needs of patients, institutions, and individuals” [15], echoed in the AAMC's [9] stance on wellbeing. Despite the longer history of the job demands and self‐determination models, systematic reviews surprisingly found that many studies of wellbeing and burnout in academic medicine in the United States, including those of faculty [22], utilized adapted instruments or a few clustered, even single, items which lack explicit theoretical grounding and justification [16, 23].

To date, little empirical evidence exists to explain the covariance, strength of effect, or hierarchy among domains of wellbeing, including both material and social contributors described as a part of organizational context, among medical school faculty. In other words, while researchers have justified the association between specific domains of wellbeing and burnout, the relative contribution of domains vis‐a‐vis each other is not well understood. Chung et al.'s [24] exploratory factor analysis study of faculty burnout is an important exception. Their focus on departmental effects took into account variance in the nature of work, goals, and professional identities, which can affect the design of wellbeing interventions. In line with faculty research overall [20], they found autonomy drives satisfaction for both clinical and basic sciences faculty, while career advancement was important for clinical faculty. However, this study did not make a link between satisfaction and burnout. While research on department type and burnout among academic medicine faculty has established descriptive differences by department [6], significant correlations between wellbeing, burnout, and control over workload are still missing. As medical schools continuously seek to integrate basic (e.g., biochemistry, physiology) and clinical sciences, such comparisons can inform greater collaboration.

Aside from the department type, scholars have highlighted the importance of personal and social characteristics on faculty's wellbeing and burnout. For instance, researchers have identified gender [5], sexual orientation [26], and racial/ethnic identities [20, 22] as important explanatory factors. Yet, disability status has not been considered in these types of studies, despite ongoing efforts for medical schools to open up to members with varying physical and emotional disabilities [27, 28].

### 1.3 Study Rationale

Due to the limitations noted in prior research, we framed our research of faculty wellbeing and burnout with a comprehensive understanding of wellbeing contributors, using Shapiro et al.'s [15] domains and sought in our analysis to interrelate various domains of wellbeing and work environment with reported burnout and control over workload. We challenge the lack of disaggregation which limits the design of interventions. This study also expands on emergent research on departmental effects [2, 6, 22] by examining potential differences in wellbeing and work environment as contributors to faculty burnout given the context of their department type and ability status. Specifically, our research addresses the following questions to identify areas that facilitate design or improvement of wellbeing initiatives for faculty in their varying contexts:

1. To what extent are faculty's wellbeing domains and work environment significantly correlated with reported burnout and perceived control over workload?
2. What are differences in faculty's experience of domains of wellbeing and work environment due to their affiliation with basic sciences, surgical, nonsurgical, and the Departments of Medicine and Pediatrics and their disability status (disability vs no disability)?
3. What are differences in faculty's experience in control over workload and frequency of burnout symptoms due to their affiliation with basic sciences, surgical, nonsurgical, and Departments of Medicine and Pediatrics, and their disability status (disability vs no disability)?

## 2 Methods

### 2.1 Data and Sample

Per the objectives of the study, the only inclusion criterion was being a faculty member at the University of Minnesota Medical School. Accordingly, all medical school faculty members (*N* = 3643) were invited to respond to the survey, of whom 703 provided complete responses. Note that 563 respondents were needed to represent an adequate sample with a 99% confidence level and 5% margin of error (i.e., the true estimate is 5% ± the obtained estimate 99% of the times), which was calculated from the online sample size calculator provided by Qualtrics^1^ based on Equation (1).

(1) $$\mathrm{Necessary}\;\mathrm{sample}\;\mathrm{size}=\frac{{\left(z-\mathrm{score}\right)}^{2}\times \mathrm{Std}\;\mathrm{dev}\times (1-\mathrm{Std}\;\mathrm{dev})}{\mathrm{Margin}\;\mathrm{of}\;{\mathrm{error}}^{2}},$$

where *Std dev* is the standard deviation indicating the extent to which individual responses vary between each other and the mean, with 0.5 being a good value to ensure obtaining a sample size that adequately represents the population of interest. The margin of error or the confidence interval represents the accuracy of the estimated sample statistic (±) from the population parameter. Finally, *z*‐score is simply the desired confidence level, with the most common percentages being 90%, 95%, and 99%. It indicates how many standard deviations from the mean is the score (i.e., raw score minus the population mean and divided by the population's standard deviation), as represented in Equation (2).

(2) $$z=\frac{\left(x-\mu \right)}{\sigma }.$$

A research team member sent the Qualtrics online survey anonymous link to *all* faculty within the University of Minnesota Medical School. The survey opened on April 17, 2022, and closed on June 7, 2022. Faculty members were asked to respond to the survey questions in addition to their department, disability status, gender, and race.

### 2.2 Research Design

The study follows a cross‐sectional research design (i.e., collecting data at a single time point). We also used a factorial analysis of variance (ANOVA) design (Disability status/Departmental affiliation) to answer the second and third research questions.

### 2.3 Measures

Inspired by Maslow's hierarchy of needs, Shapiro et al. proposed a wellbeing instrument with five domains: (a) basic needs, (b) safety, (c) respect, (d) appreciation, and (e) contribution [14]. In collaboration with subject matter experts, the research team developed a comprehensive survey based on Shapiro's work with three items for basics (e.g., I have enough personal time), four for safety (e.g., I have job security), five for respect (e.g., I feel respected by my peers), four for appreciation (e.g., I feel appreciated by my peers), and two for contribution (e.g., I am given the opportunity to provide input regarding institutional decisions). We also added a domain on work environment with seven items measuring the supportiveness of the environment for wellbeing (e.g., Someone in our organization cares about my wellbeing and is working to improve it). We also added one item to assess perceived control over workload (e.g., My control over my workload is…) and another on frequency of burnout symptoms (e.g., The symptoms of burnout (emotional exhaustion, depersonalization, reduced personal efficacy) to identify the correlation, if any, among wellbeing domains, work environment, burnout, and control of workload. All survey items were rigorously reviewed by subject matter experts and the researcher team to collect validity evidence based on the survey content. Relatedly, all survey scales had adequate score reliability estimates, since estimates were 0.72, 0.80, 0.85, 0.87, 0.72, and 0.61 for the basic needs, safety, respect, appreciation, contribution, and work environment, respectively.

The wellbeing domains and work environment survey items used a five point rating scale ranging from *strongly disagree* (1) to *strongly agree* (5). The higher the score, the greater the feeling that basic needs are satisfied, greater the perception of safety, feeling more respected and appreciated, greater contribution to the institution, as well as the supportive nature of the working environment. For each domain, we computed a sum score to be utilized in the statistical analyses as described later.

The control over workload item included five response options but with different labels ranging from *poor* (1) to *optimal* (5). On the other hand, six response options were utilized in assessing the frequency of burnout symptoms ranging from *never* (0) to *every day* (5). The higher the number, the greater the perceived control over workload and frequency of burnout symptoms, respectively.

### 2.4 Data Analysis

We analyzed deidentified data in line with the three research questions listed above. First, Pearson correlation coefficients were estimated among the five domains and work environment, with higher scores indicating that these domains were better met. Kendall's coefficient of rank correlation was used with the burnout and control over workload, given their ordinal nature [29]. To identify potential areas for interventions that could help improve the domain factors for those most affected, we sought areas with strong associations for practical significance. Alternatively, to assess the practical significance of the estimated correlation coefficients, we utilized Cohen's 1988 [30] guidelines with |*r*| < 0.30, 0.30 ≤ |*r*| ≤ 0.50, and |*r*| > 0.50, indicating a weak, moderate, and strong association, respectively. Second, we conducted a two‐way ANOVA followed by a post hoc analysis to test for the differences in faculty wellbeing domains due to their self‐reported department type and disability status. Departments were clustered by work type with two large nonsurgical departments (Medicine and Pediatrics) analyzed separately. The remaining nonsurgical departments were clustered as “non‐surgical departments” or basic sciences.

To adjust for the multiple comparisons for the department type factor and control family‐wise error rates, we utilized Bonferroni's adjustment method. We also quantified the practical significance for the statistically significant results using partial eta‐squared following Cohen's 1988 guidelines, where values of 0.01, 0.06, and 0.14 are small, moderate, and large, respectively. Third, we fitted a two‐way ordinal ANOVA model with a cumulative link, since burnout and control over workload were each assessed by only one ordinal item. All analyses were conducted using *R* (R Core Team, 2023). As the data are normative (e.g., peer comparisons between departments and disability), potential differences between these groups indicate that wellbeing domains and work environment are norm‐referenced differences. This study was approved by the Internal Review Board (IRB) of the University of Minnesota Medical School.

## 3 Results

Of the 703 faculty members who provided complete responses, the nonsurgical cluster of departments had the most respondents (*N* = 235), followed by the Department of Medicine (*N* = 146; see Table 1 for more information on department clustering). Regarding disability status, 670 faculty members reported having no disability, whereas 33 respondents reported having a disability.

**Table 1:** Department clustering and sample size.

Department cluster	Departments	*N*
Basic Sciences	Biochemistry, Molecular Biology and Biophysics; Biomedical Sciences, Genetics – Cell Biology and Development, Integrative Biology and Physiology, Microbiology and Immunology, Neuroscience, Pharmacology	77
Department of Medicine	Medicine	146
Non‐surgical	Dermatology, Emergency Medicine, Family Medicine and Bio‐behavioral Health, Family Medicine and Community Health, Radiation Oncology, Laboratory Medicine and Pathology, Neurology, Psychiatry and Behavioral Sciences, Radiology, Rehabilitation Medicine	235
Department of Pediatrics	Pediatrics	104
Surgical	Anesthesiology, Neurosurgery, Obstetrics Gynecology and Women's Health, Ophthalmology and Visual Neurosciences, Orthopedic Surgery, Otolaryngology, Surgery, Urology	141

Statistical results are reported in sequence with the three research questions listed above. For the first, as shown in Table 2, wellbeing domains were all positively and significantly correlated with one another (*p* < 0.001), with the largest correlation observed between appreciation and respect (*r* = 0.837), whereas the smallest correlation was observed between appreciation and basics (*r* = 0.507). As expected, burnout was negatively and significantly correlated with the five domains, work environment, and control over workload, indicating higher levels of burnout were associated with lower levels of wellbeing and support from the work environment. Additionally, results indicated that higher levels of wellbeing were associated with higher levels of control over workload. All correlations were moderate to strong according to Cohen's guidelines.

**Table 2:** Correlation matrix among the wellbeing domains, burnout, and control over workload.

Variables	*M* (SD)	Basics	Safety	Respect	Appreciation	Contribution	Work environment	Burnout	Workload
Basics	10.8 (2.58)	1							
Safety	14.85 (3.17)	0.663	1						
Respect	19.52 (3.81)	0.616	0.749	1					
Appreciation	15.6 (3.54)	0.507	0.735	0.837	1				
Contribution	6.65 (1.97)	0.575	0.644	0.637	0.611	1			
Work environment	22.43 (4.69)	0.633	0.717	0.686	0.680	0.640	1		
Burnout	‐‐‐	−0.625	−0.593	−0.528	−0.515	−0.513	−0.634	1	
Control over workload	‐‐‐	0.635	0.560	0.456	0.404	0.550	0.588	−0.566	1

*Note:* “‐‐‐” indicates that these two variables were assessed by an ordinal item each, so no means or standard deviation were computed. *M* and SD refer to means and standard deviations, respectively. All correlations are statistically significant at *p* < 0.001.

For the second research question, Table 3 shows the means and standard deviations of the wellbeing domains as a function of the department type and disability status. Descriptively, faculty in the basic sciences departments reported, on average, higher levels of wellbeing compared to other departments, particularly the surgical departments. In addition, those without disability descriptively reported higher scores, on average, compared to those with disability.

**Table 3:** Means (standard deviations) for the wellbeing domains and work environment as a function of department type (basic sciences, medicine, nonsurgical, pediatrics, surgical) and disability status (no, yes).

Department types	Disability status	Wellbeing domains					
		Basics	Safety	Respect	Appreciation	Contribution	Work environment
Basic Sciences	No (*n* = 70)	11.88 (2.37)	15.00 (3.10)	20.23 (3.33)	15.46 (3.20)	7.09 (1.67)	23.93 (4.82)
	Yes (*n* = 7)	10.57 (2.15)	14.00 (3.00)	17.86 (4.49)	12.71 (4.23)	6.71 (1.98)	18.33 (4.32)
Department of Medicine	No (*n* = 139)	10.62 (2.42)	14.84 (3.01)	19.25 (3.68)	14.68 (3.55)	6.33 (2.12)	22.12 (4.79)
	Yes (*n* = 7)	10.29 (2.50)	14.86 (3.44)	20.29 (4.15)	15.43 (3.26)	5.86 (2.41)	22.14 (7.06)
Nonsurgical	No (*n* = 223)	11.20 (2.42)	15.16 (3.15)	20.13 (3.69)	15.53 (3.62)	6.92 (1.97)	22.85 (4.53)
	Yes (*n* = 12)	11.00 (2.59)	16.25 (3.36)	21.08 (4.03)	15.25 (3.93)	7.00 (2.13)	22.08 (5.37)
Department of Pediatrics	No (*n* = 100)	10.76 (2.30)	14.92 (3.21)	19.66 (3.47)	15.64 (2.98)	6.74 (1.87)	22.32 (4.70)
	Yes (*n* = 4)	8.75 (2.22)	11.50 (5.07)	16.25 (3.30)	12.50 (4.12)	5.00 (1.15)	18.25 (3.40)
Surgical	No (*n* = 138)	10.31 (2.86)	14.67 (3.05)	18.84 (4.04)	15.02 (3.66)	6.42 (1.84)	21.97 (4.26)
	Yes (*n* = 3)	7.00 (3.61)	12.00 (3.46)	18.00 (4.58)	12.67 (3.21)	6.00 (1.00)	16.33 (4.16)

*Note:* Standard deviations are between parentheses.

Turning to the two‐way ANOVA results as shown in Table 4, we found a statistically significant effect of disability status on having basic needs satisfied, *F*_(1, 701)_ = 4.72, *p* = 0.03,η ^2^ = 0.01 and work environment, *F*_(1, 701)_ = 7.90, *p* < 0.01,η ^2^ = 0.01, indicating that fewer faculty who reported a disability have basic needs met and felt supported by their work environment than those without a disability. Department type had a statistically significant effect on three domains: basics, *F*_(4, 698)_ = 6.17, *p* < 0.001,η ^2^ = 0.03; respect *F*_(4, 698)_ = 3.40, *p* < 0.01,η ^2^ = 0.02; and contribution, *F*_(2, 698)_ = 3.68, *p* < 0.01,η ^2^ = 0.02. For safety and appreciation, there was no statistically significant effect, neither for the department type nor disability status.

**Table 4:** Two‐way ANOVA results for the wellbeing domains and work environment as a function of department type (basic sciences, medicine, nonsurgical, pediatrics, surgical) and disability status (no, yes).

Wellbeing domain	Factor	Sum of squares	Mean of squares	*F*	Partial Eta‐squared
Basics	Department	154.04	38.51	**6.17** ^***^	0.03
	Disability	29.47	29.47	**4.72** ^*^	0.01
Safety	Department	35.8	8.95	0.91	‐‐‐
	Disability	7.03	7.03	0.72	‐‐‐
Respect	Department	188.56	47.14	**3.40** ^**^	0.02
	Disability	5.32	5.32	0.38	‐‐‐
Appreciation	Department	74.52	18.63	1.52	‐‐‐
	Disability	38.52	38.52	3.14	‐‐‐
Contribution	Department	55.04	13.76	**3.68** ^**^	0.02
	Disability	5.09	5.09	1.36	‐‐‐
Work environment	Department	184.76	46.19	2.15	‐‐‐
	Disability	169.61	169.61	**7.90** ^**^	0.01

*Note:* *, **, and ***, respectively, indicate significance levels or *p* < 0.05, 0.01, and 0.001.

To determine any statistically significant effect of the department type, we conducted post hoc multiple comparisons between each compared pair of the department type (see Table 5). Results revealed that faculty in basic sciences departments had statistically higher scores on the basic needs domain compared to the surgical departments, and the departments of Medicine and Pediatrics. Basic sciences departments also had higher scores on the contribution domain compared to those affiliated with the Department of Medicine. Additionally, faculty in the nonsurgical departments reported statistically significant higher scores on the basics and respect domains compared to those affiliated with the surgical departments. They also reported higher scores on the contribution domain compared to faculty in the Department of Medicine.

**Table 5:** Post hoc results for the wellbeing domains and work environment as a function of department type (basic sciences, medicine, nonsurgical, pediatrics, surgical).

Compared departments	Wellbeing domains					
	Basics	Safety	Respect	Appreciation	Contribution	Work environment
Medicine – Basic Sciences	−**1.20** ^**^	−0.09	−0.73	−0.54	−**0.76** ^*^	−1.43
Nonsurgical – Basic Sciences	−0.61	0.29	0.15	0.26	−0.14	−0.73
Pediatrics – Basic Sciences	−**1.13** ^*^	−0.15	−0.51	0.24	−0.40	−1.41
Surgical – Basic Sciences	−**1.59** ^***^	−0.32	−1.22	−0.32	−0.67	−1.77
Nonsurgical – Medicine	0.59	0.37	0.88	0.80	**0.62** ^*^	0.69
Pediatrics – Medicine	0.07	−0.06	0.22	0.79	0.36	0.02
Surgical – Medicine	−0.38	−0.24	−0.49	0.22	0.09	−0.34
Pediatrics – Nonsurgical	−0.52	−0.43	−0.65	−0.01	−0.26	−0.68
Surgical – Nonsurgical	−**0.98** ^**^	−0.61	−**1.37** ^**^	−0.58	−0.53	−1.04
Surgical – Pediatrics	−0.46	−0.18	−0.72	−0.56	−0.27	−0.35

*Note:* Mean differences estimates are reported, where *, **, and ***, respectively, indicate significance or at *p* < 0.05, 0.01, and 0.001.

For the third research question, results of the two‐way ordinal ANOVA model revealed that the department type and disability status significantly affected the frequency of burnout, where *χ* ^2^_(4)_ = 18.86, *p* < 0.01 and *χ* ^2^_(1)_ = 4.34, *p* < 0.05, respectively, indicating that faculty with disability frequently experienced higher levels of burnout, on average, compared to faculty without disability. For the departmental effect, results of multiple comparisons revealed that faculty at the basic sciences departments reported lower levels of burnout compared to other departments. Regarding the perceived control over workload, results were not statistically significant for both the department types (*χ* ^2^_(4)_ = 3.28, *p* = 0.51) and disability (*χ* ^2^_(1)_ = 1.76, *p* = 0.18).

## 4 Discussion

Most strikingly, being part of a basic sciences department and not reporting a disability was associated with lower frequencies of burnout than being in clinical departments, especially surgical departments, or reporting a disability. While we cannot determine exactly why this burnout is occurring since our data were correlational, not as casual as those obtained from an experimental design (i.e., a randomized control trial where external variables are controlled), our findings point to some emergent links among domains of wellbeing, work environment, and control over workload. These correlational links do merit further investigation and potential changes to pilot and evaluate in wellbeing reforms and fill in the associations sketched out in the literature between domains of wellbeing and work environment as well as wellbeing and control over workload.

Results confirmed the hierarchical nature of Shapiro's model because basic needs have the strongest negative correlation with burnout, and in each successive level, the strength of the correlation diminishes. However, when designing interventions, it is important to note that associations among the domains may not be step‐wise. For instance, respect is even more strongly correlated with other domains than basic needs being met. This suggests the fundamentality not only of material needs, but an inherently social domain (respect), to wellbeing. This finding aligns with critiques in the literature of more individualistic readings of psychological needs from self‐determination theory [13]. Even though respect itself does not have the strongest correlation with burnout, given its correlations with other domains, respect may itself be a mediating factor to other domains or evidence that these other domains are (or are not) being met at the department level.

Current results also confirmed that control over workload, a type of autonomy, remains important [6, 20] to faculty wellbeing and burnout, and their association is somewhat complex. Notably, control over workload does not have the strongest correlation with burnout, which means that it alone may not be a sufficient way to potentially mitigate burnout or explain its effects. Since faculty with disabilities experience a higher frequency of burnout but ability status does not explain differences in control over workload, researchers and practitioners may look to other factors for possible explanations or solutions. We also learned that basics, safety, and contribution have stronger associations to control over workload than respect or appreciation. While it is not definitive if having basic needs met, safety, and contribution can facilitate control over workload or vice versa, this schema can help more thoroughly and actionably describe feelings of autonomy that negatively correlate with burnout.

Diving deeper into the work environment and control over workload, more commonly represented in the literature, via wellbeing domains is critical as our data show departments cannot easily be proxies for all differences in experience. While work environment and control over workload did not vary significantly among specific departments, basic needs, respect, and contribution did, as did the frequency of burnout. Notably, while most current studies [22, 24], characterize the supportiveness of the work environment in terms like those in basic needs and appreciation, safety was even more strongly correlated in our study to work environment. Fewer faculty with disabilities indicate their basic needs are met and they feel supported by their work environment. Thus, researchers and medical educators should consider the connection of safety with basic need fulfillment and work environment and what these bring to bear on faculty with disabilities' experiences. Differences in safety and perceived supportiveness of the work environment, according to our data, cannot be explained by the department. This begs the question if the work environment, especially for faculty with disabilities, could be due to institutional factors or something else outside of the departments' mission, foci, and leadership.

A specific mission focus above a department is medical schools' drive to integrate basic and clinical sciences, now housed in separate departments, via collaboration between these departments in curriculum design and teaching [25]. Thus, higher levels of burnout among faculty in clinical departments and variations in basic needs, respect, and contribution by department type (e.g., Medicine vs. surgical) could indicate potential tensions or barriers to collaboration between and among basic sciences and clinical faculty. The current study revealed some of these types of differences, though was limited in explaining implications for collaboration or why they exist due to design. Overall, basic sciences departments scored higher on the basics than surgical departments, the departments of Medicine and Pediatrics, but not the other nonsurgical departments. This may not be surprising given the physical and time demands of patient care. Yet, given the importance of basics to other domains of wellbeing as well as to burnout itself, interventions that fail to acknowledge this difference may not deliver. Surgical departments also scored lower in both basic needs being met and respect, the two foundational domains highly correlated with other wellbeing domains, than nonsurgical departments as well as basic sciences departments. Thus, basic need fulfillment may not be limited to the difference in job functions between clinical and nonclinical faculty.

### 4.1 Limitations

The results of the current study should be generalized with caution, given some limitations. Most importantly, data are from one institution, and the study design is correlational, so future research can replicate the study using data from more than one medical school, utilizing other research designs (e.g., randomized control trials) to allow for drawing causal inferences. In addition, qualitative research could elucidate why departmental differences indicated in this study may exist and the implications of these differences for potential collaboration in and between department types. Finally, data are self‐reported, and subjective differences in interpreting the study measures beyond their given definitions in the instrument (e.g., respect) could influence the study results.

### 4.2 Implications for Research and Practice

The analysis of wellbeing models empirically with specific faculty populations, notably departments, can result in more efficient and effective measures and, thus, the design of wellbeing interventions. While previous research studied faculty wellbeing and burnout as one domain (e.g., contribution) or conceptually gave equal weight to various domains' influence on burnout [15], this research confirmed the importance of conceptually distinguishing domains as well as carefully selecting which have the strongest links to burnout. In other words, domains of wellbeing are essential but are not created equal, at least in the context of the medical school studied. Furthermore, differences in key domains among departments indicate the need for evaluation and potentially prioritization of wellbeing initiatives. For instance, because surgical departments scored lower than nonsurgical clinical departments and basic sciences departments in both basic needs being met and respect, basic need fulfillment may not be just a in clinical and nonclinical job functions. In addition, an emphasis on the contribution domain for surgical departments may not target the root of higher levels of burnout. Thus, additional follow up to determine why surgical faculty lack in basics compared with other clinical faculty and where differences in respect emerge could inform policy and resource allocation changes. With the disaggregation not only by department type and faculty characteristic but also in the construct of wellbeing itself, this research offers to medical schools the opportunity to more specifically plan and evaluate inclusivity and wellbeing initiatives.

## 5 Conclusions

Medical schools have historically assessed faculty wellbeing without considering the potential weight and covariance of measures correlated with burnout—a critical issue in the field. As medical schools assess faculty wellbeing and evaluate related interventions and evaluating the effectiveness of wellbeing interventions at the organizational level to reduce burnout, they should also consider disaggregating data by domain, department, and ability status given current results. Further research should investigate why perceived basic needs being met, respect, and contribution are consistently higher or lower across departments and ability status, whereas other contributors to wellbeing linked with mitigation of burnout, such as safety and appreciation are not. Future wellbeing evaluations or intervention designs may also consider how the work environment or control over workload may involve issues of safety as well as basics and contribution. Tailoring wellbeing efforts to prioritize and even cluster specific domains more strongly correlated with burnout and each other, especially for faculty with disabilities or in surgical departments, could ultimately impact faculty's direct contributions to the healthcare system and their students' learning.

## Author Contributions

**Mohammed A. A. Abulela:** conceptualization, writing–original draft, writing–review and editing, formal analysis, software, methodology, project administration, validation, investigation, supervision. **Bethany Schowengerdt:** conceptualization, writing–original draft, writing–review and editing, investigation. **Heather Dorr:** writing–review and editing, data curation. **Amanda Termuhlen:** conceptualization, project administration. **Kristina Krohn:** writing–review and editing. **Claudio Violato:** writing–review and editing, project administration, supervision.

## Ethics Statement

The study was conducted according to the ethical principles and standards for conducting Medical Research, and it was approved by the Internal Review Board (IRB) of the University of Minnesota Medical School.

## Conflicts of Interest

The authors declare no conflicts of interest.

### Transparency Statement

The lead author as well as all co‐authors affirm that this manuscript is an honest, accurate, and transparent account of the study being reported; that no important aspects of the study have been omitted; and that any discrepancies from the study as planned (and, if relevant, registered) have been explained.

## Data Availability

This is a health sciences project, and data are not available.

## Endnotes

1 Qualtrics sample size online calculator and associated equations can be obtained from this link https://www.qualtrics.com/blog/calculating-sample-size/.
